# Does hard mast production affect patterns of cementum annuli formation in premolar teeth of Asian black bears (*Ursus thibetanus*)?

**DOI:** 10.1371/journal.pone.0211561

**Published:** 2019-02-04

**Authors:** Kahoko Tochigi, Yukino Aoki, Tetsuya Maruyama, Koji Yamazaki, Chinatsu Kozakai, Tomoko Naganuma, Akino Inagaki, Takashi Masaki, Shinsuke Koike

**Affiliations:** 1 Tokyo University of Agriculture and Technology, Fuchu, Tokyo, Japan; 2 Tochigi Prefectural Forestry Research Center, Utsunomiya, Tochigi, Japan; 3 Tokyo University of Agriculture, Setagaya, Tokyo, Japan; 4 National Agriculture and Food Research Organization, Tsukuba, Ibaraki, Japan; 5 Forestry and Forest Products Research Institute, Tsukuba, Ibaraki, Japan; Ecole Normale Supérieure de Lyon, FRANCE

## Abstract

Cementum annuli widths in mammals are is influenced by the nutrition of mammals. Reproductive stress has been is suggested to reduce the width of lead to narrower cementum annuli widths in female Asian black bears (*Ursus thibetanus*); however, food availability in autumn strongly impacts bear nutrition and likely impacts cementum widths as well. This study aimed to test how cementum annuli widths and the formation of false annuli were influenced by hard mast production. We established two hypotheses: (1) cementum annuli widths become narrower in poor mast years owing to inadequate nutritional conditions and (2) false annuli occur more frequently in poor mast years. We used teeth samples from male bears to avoid reproductive influences and separated width data into “adult” and “subadult” groups. We calculated the proportional width index (PWI) and used linear mixed models to estimate the masting effects on PWI. Generalized linear mixed models estimated the masting effects on false annuli frequency. True annuli widths and false annuli formation showed no significant relationship with mast production in adults. In subadults, poor mast production weak negative influence on false annuli formation. These new data resolve previous questions, allowing us to deduce that cementum annuli widths are a reliable index of reproductive success in female bears.

## Introduction

**Fig 1:**
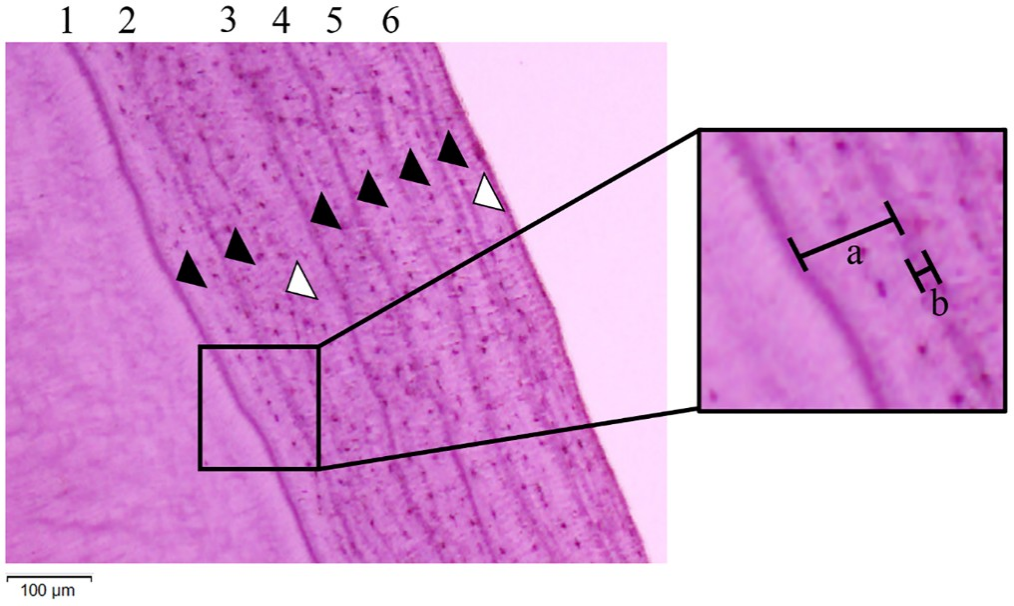
Photomicrograph of cementum annuli in the first premolar tooth of a male Asian black bear (ID = 22–12). Numbers above the photomicrograph are age in years; this bear was estimated to be 6 years old. Black arrows indicate annuli, and white arrows show false annuli (faint or non-continuous dark bands). Band “a” in the panel at right corresponds to a light band and band “b” is a dark band.

For some organisms, banded growth lines on specific body parts document periodic growth [1]. For example, the growth line, or annual ring, is produced in the xylem of some woody plant species representing a single year’s growth and often is used as an estimate of tree age. Like woody plants, some mammal species have growth lines that form in a periodic manner on the cementum of their teeth called cementum annuli [1]. Cementum is connective tissue that grows in incremental layers surrounding the roots of teeth, becoming thick with mineral deposition [2]. These bands consist of both thick light-stained bands (hereafter, light band) and narrow, dark lines (hereafter, dark band) (Fig 1).

Numerous studies have shown that cementum annuli are present in a number of mammal species, and it is possible to estimate the age of an individual by counting the cementum annuli (terrestrial mammals in [3]; marine mammals in [4]). Additionally, by clarifying the periods that the light bands and dark bands were created, it is possible to estimate the season of death from the tooth of a carcass [5]. Moreover, in the study of cementum annuli, researchers must be aware of false annuli, which are known to be produced as a result of rapidly changing growth rates, behavioral changes, or stress caused by mating, bad weather, or unseasonable temperatures [6].

Various factors influence cementum annuli widths. It decreases with age and stops decreasing at sexual maturity; the pattern of decrease often corresponds to the pattern of body growth [1]. Cementum annuli widths increase during years when an animal’s diet is higher in nutrients [2]; however, cementum deposition is disrupted during pregnancy and lactation [7, 8], such that annuli widths in female teeth become narrower in years when offspring are delivered and lactation occurs, as the females relinquish nutrition to feed their young [9–11]. Therefore, the factors that influence changes in annuli widths differ between males and females.

The cementum annuli of Asian black bears (*Ursus thibetanus*) have been used for many years to provide an estimation of age [12, 13], although there is little fundamental knowledge about the accumulation of cementum in bears. Our most recent study shows that the cementum annuli in female Asian black bears are reflective of their reproductive histories, as widths become narrower owing to poor nutritional conditions during lactation [14]. However, bear nutrition in relation to food resource availability was not considered in that study. In general, female bears are likely to give birth in years following an autumn of good hard mast production; the years they give birth and raise the cubs tend to be poor mast years because mast production alternates each year [15]. Thus, annuli widths in female bears regularly become narrower in the years when they successfully reproduce, but the availability of food resources is also low during that period, affecting their nutritional condition. This makes it difficult to separate the true factors affecting annuli widths: reproductive success or low food resource availability.

The first objective of our study was to test how the widths of cementum annuli are affected by fluctuating nutritional conditions owing to changes in hard mast production in autumn each year. The level of hard mast production has an impact on the nutritional status of the American black bear (*Ursus americana*) [16]. Additionally, because bears, and other mammals, produce false annuli (faint or separated dark bands; Fig 1) in the cementum owing to stressful events [1, 9], our second objective was to test whether the formation of false annuli was likely to be affected by hard mast production. We established two hypotheses corresponding to the above two objectives: (1) cementum annuli widths become narrower owing to bad nutritional conditions during poor mast years and (2) false annuli occur more frequently in poor mast years.

To clarify these hypotheses, firstly, it was necessary to identify the seasons during which light bands were formed. Poor nutritional conditions owing to lactation in the spring and summer have an impact on cementum annuli widths [9], but hard mast is produced and eaten by bears during late summer and autumn [17]. Thus, by clarifying the periods of light-band formation, it would be possible to correlate the nutritional conditions for which period affected light-band formation versus false annulus formation, and then verify the effects of hard mast production on width. Secondly, we investigated how cementum annuli widths were influenced by hard mast production in current years to clarify the factor that most strongly influenced cementum annulus formation with the goal of determining whether cementum annuli could be used as a reliable indicator of reproductive success in bears. We also estimated the influence of hard mast production in autumn on cementum annuli width in the following year, as bears can survive and grow in the following year by using energy from the fat accumulated in previous periods of hyperphagia [18]. Third, by using relative width values [19], we determined whether the pattern of decrease in cementum annuli width showed a trend similar to those seen in other mammals [1]. The index cannot be calculated without confirming if the cementum annuli widths of bears show a decreasing pattern with age.

We used data from the annuli widths of adult male and subadult male bears because these bears do not give birth and raise offspring, so they would not be influenced by nutritional deficiencies due to reproduction. However, in our analyses, we separated these data into adult and subadult periods because there are differences in nutritional conditions related to cementum deposition between adult and young bears [20]. In Japan, prefectural governments manage bears; in the study area has a Specified Wildlife Conservation and Management Plan that includes a plan for the control of bears. Bear control is conducted legally; the annual bag limit for hunting or killing is set at 15% of the median estimated abundance in the study area [21]. This percentage is based on bear numbers and distribution and human–bear conflict. Killing must target only those bears liable to cause harm to humans, as well as bears not affected negatively by relocation and bears that have become used to humans [21].

## Materials and methods

### Treatment of bear teeth

We used the first premolar teeth from 85 male bears killed, a method that minimizes as much as possible the pain and distress to the animal shall be used in accordance with “Welfare and Management of Animals Act” (Ministry of the Environment), because of nuisance behavior such as trying intrude human settlement in Tochigi Prefecture, Central Japan, from 2007 to 2014. These captures were conducted under the permission obtained for the present study from the Tochigi Prefecture Government according to “Wildlife Protection and Proper Hunting Act” (Ministry of the Environment) and “Specified Wildlife Conservation and Management Plan” (Tochigi Prefecture Government), which covered the controlled killing of bears. Permission numbers of the Tochigi Prefecture Government permits are 19–8, 19–13, 19–36, 19–37, 20–4, 20–10, 20–31, 20–35, 21–1, 21–10, 21–16, 22–6, 22–11, 22–12, 22–20, 22–21, 22–25, 22–27, 22–28, 22–40, 22–42, 22–43, 22–55, 23–6, 23–7, 23–10, 23–11, 23–16, 23–18, 23–19, 23–20, 23–25, 23–30, 24–5, 24–9, 24–11, 24–12, 24–13, 24–16, 24–18, 24–19, 24–25, 24–30, 24–33, 24–37, 24–44, 24–45, 24–46, 24–48, 24–49, 24–50, 24–52, 24–53, 25–2, 25–4, 25–12, 25–14, 25–15, 25–16, 25–24, 26–2, 26–3, 26–24, 26–29, 26–78. In our study area, euthanasia is selected mainly to kill bears. Bears are euthanized as soon as possible after capture; a shotgun is used, so that the cerebral hemispheres and brainstem are sufficiently disrupted by the projectile to induce immediate loss of consciousness and subsequent death [22].

We used a total of 85 bears to identify the periods of cementum annuli formation for estimating ages and 64 bears to analyze the relationship between cementum annuli widths and hard mast production. The dataset was divided into two groups, namely adult period (more than 3 years old; n = 29) and subadult period (1 to 3 years old; n = 51), on the basis of age at the time of sexual maturation [23]. We subtracted the width data from 0–1-year-old bears as well as the width data from the far-most ends of the annuli. The period of permanent tooth development and cementum deposition for the youngest bears may differ among individuals and the far-most ends of the annuli would still be forming. However, it is not verified if there is difference and how much different is annuli width among these young individuals. Thus these differences cannot be compensated for by an index of cementum annuli widths, as detailed below. The same individuals were used in both the adult and subadult periods because we separated the data by the age of formation for each cementum annulus from all the teeth collected. For example, for an individual that was seven years old, we classified the cementum width data from 1–3 years into the subadult period and the data from 4–6 years into the adult period.

We treated the teeth using the methods of Ohtaishi et al. [24]. The collected first premolar teeth were soaked in Plank–Rychlo’s decalcification solution for 13 h, washed for 1 h, and then neutralized in a sodium hydroxide aqueous solution for 4 h. Afterward, the roots were cut by a microtome (REM-710, Yamato Kohki Industrial Co., Ltd., Asaka, Japan) into sections with a thickness of 20 μm. For each tooth, eight sections were sliced and mounted on a glass slide. Sections were stained with Carrazzi’s hematoxylin solution for 2 h and photographed using a mounted camera (DP21, OLYMPUS, Tokyo, Japan) on a transmitted light microscope (CX41, OLYMPUS).

False annuli are often observed in teeth from both males and females, where dark bands appear broken or light-colored, making it impossible to distinguish a true annulus from a false annulus [11, 25]. We avoided counting false annuli and, instead, estimated age using eight sections for verification of the masting effects on cementum annuli widths. We also used four of those eight sections to measure the cementum widths. In addition, we recorded the presence of false annuli for each annuli width to verify the masting effects on false annuli formation.

### Identifying periods for cementum annuli formation

We observed the edges of the cementum annuli from all individuals (n = 85; Table 1) to determine when light bands and dark bands were formed for evaluating our first hypothesis.

**Table 1:** Capturing month of individual Asian black bears during 2007–2014 in Tochigi Prefecture.

Month of capture	No. of bears
Apr.	1
May	2
Jun.	4
Jul.	11
Aug.	24
Sep.	15
Oct.	24
Nov.	3
Dec.	0
Jan.	1
Feb.	0
Mar.	0
Total	85

### Relationship between cementum annuli widths and hard mast production

The hard mast crop size was investigated by visual assessments of seed production for *Quercus crispula* in early September from 2006 to 2013 in the Ashio-Nikko Mountains, Tochigi Prefecture [14, 26]. *Q*. *crispula* is the dominant hard mast species and staple food for bears; only the masting of *Q*. *crispula* influences bear behavior in this area, even though there are other hard mast species present with annual changes in mast production [27]; therefore, we calculated the energy value of each *Q*. *crispula* tree and averaged them for each year during the study period.

First, we measured the cementum annuli widths (*x*_*i*_) for each age *i* from all individuals without separating the light and dark bands. In each of the eight sections, we used imaging software (cellSense, Olympus) to take 20 measurements at intervals of 50 μm; thus, the number of data points recorded per annulus for each individual was 160. After the measurements had been taken, we averaged the 160 data points, calculated proportional growth layer group width (PW) [19], and surveyed the relationship between PW and age when the *i* -th annulus was formed. PW considers the variation in cementum deposition that may occur between individuals (varying tooth sizes) or the variation due to aging, and is expressed as

PWi=xi∑0ixi

PW0=x0∑01x0 (1)

Second, we calculated the proportional width index (PWI) using the above-determined PW to eliminate these effects [19]. Specifically, PWI compares the PW for each age cohort to the mean for all individuals of that age, creating a relative index of growth that is independent of age. This is accomplished by dividing individual PWs by mean PWs for all ages *i* (PW_*im*_) and then performing a log transformation of the data.

PWIi=log10(PWiPWim+1) (2)

We used segment linear regression to make sure whether the decreasing patterns of cementum annuli widths showed a tendency similar to that seen in other mammals [1]. We set PW as the response variable and age as explanatory variable, and estimated break point by the segmented package [28].

We used linear mixed models (LMMs) because PWI is supposed to follow a normal distribution [19]. To clarify whether decreased cementum annuli widths in this study were associated with poor hard mast production, we set PWI as the response variable, whereas the explanatory variables were taken from the log-transformed dataset of averaged hard mast energy values from previous or current years when the annuli were formed. Bears may survive and grow from winter to the following autumn using only the energy that they accumulated when feeding on hard mast production the previous autumn; thus, we thought that previous year masting may have an effect on current year cementum annuli widths. In contrast, the current year masting model was also developed because light bands may form until autumn and are then influenced by the accumulation of energy in the current autumn season. Individual IDs were defined as random effects. We used the lme4 package [29] within R 3.3.0 [30] to create the models and estimate the effects of hard mast production. We also created a null model and made comparisons across six different models (previous masting model, current masting model, and null model for the adult group and the subadult group) to determine whether the estimated effects of hard mast production on cementum annuli formation were significant, and then which effects were significant for the previous year or current year. The relative model fit was examined using Akaike’s information criterion (AIC) statistics [31], where the lowest AIC value indicates the best-fit model.

### Relationship between false annuli and hard mast production

We used generalized linear mixed models (GLMMs) for the previous and current year masting models for both the adult and subadult groups to determine whether the frequency of false annuli increased owing to poor masting. We replaced PWI values with false annuli data (0, 1) as the response variable. The probability distribution followed a binominal distribution pattern, and individual IDs were defined as random effects. We used the lme4 package [29] to create models, and the relative model fit was examined using AIC.

## Results

Until September, light bands were formed in the teeth of all individuals, and no dark bands were formed. Formation of dark bands was observed in October (in 8 of 24 bears), November (0 of 3), and January (1 of 1) (Table 1).

We selected the 64 bears and estimated age to analyze the relationship between cementum annuli widths and hard mast production. The most common age of the bears in our study was 5 years followed by 6 year-olds (Table 2).

**Table 2:** Age structure of individual Asian black bears for statistical analyzes during 2007–2014 in Tochigi Prefecture.

Age	No. of bears
2	2
3	6
4	5
5	21
6	12
7	5
8	6
9	3
11	1
13	1
14	1
15	1
Total	64

**Fig 2:**
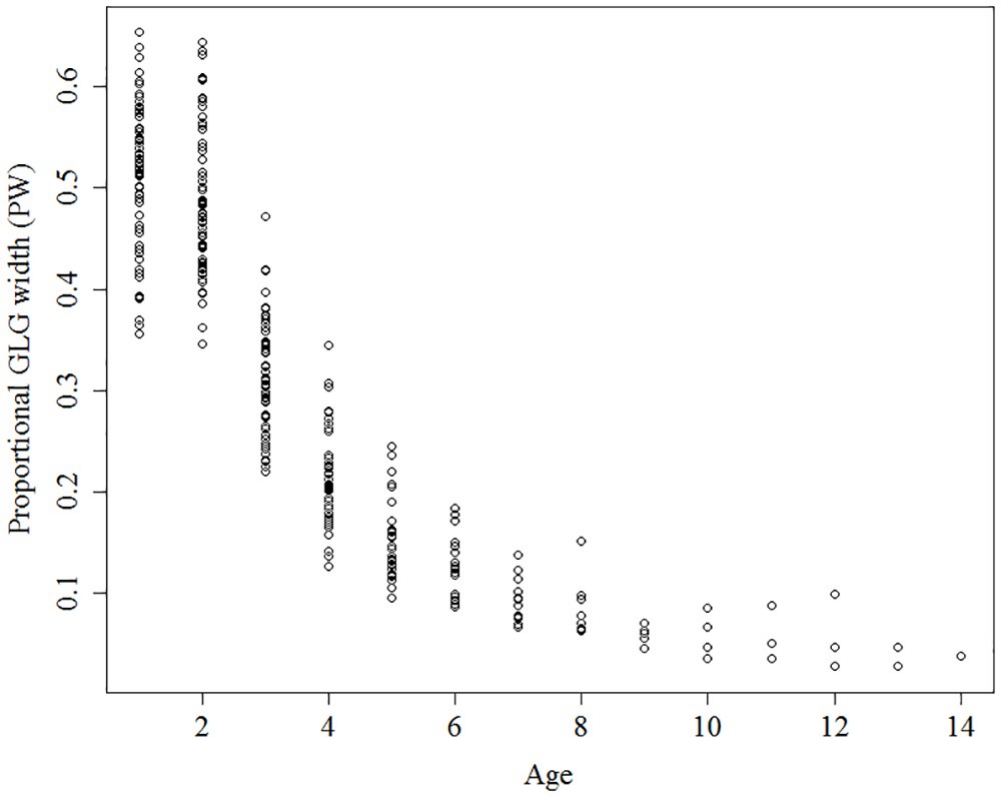
Relationship between age of Asian black bears and proportional growth layer group width (PW).

We confirmed the decreasing pattern in cementum formation using PW values that were calculated in the process of determining PWI. Decreases in cementum annuli widths were observed with increases in age (Fig 2). The results of the segmented linear regression indicated that the “break point” for a male bear is 3.7 years old (SE = 0.15), such that decreasing annuli widths occurred only to 3.7 years of age in male bears.

We found that the false annuli formed accounted for 52.2% of the total number of annuli (adult = 40.3%, subadult = 57.1%). In addition, we determined that the ratio of false annuli to total annuli was significantly different between adults and subadults using the Z-test for the equality of two proportions (*X*^*2*^ = 4.94, *df* = 1, *P* = 0.03).

**Fig 3:**
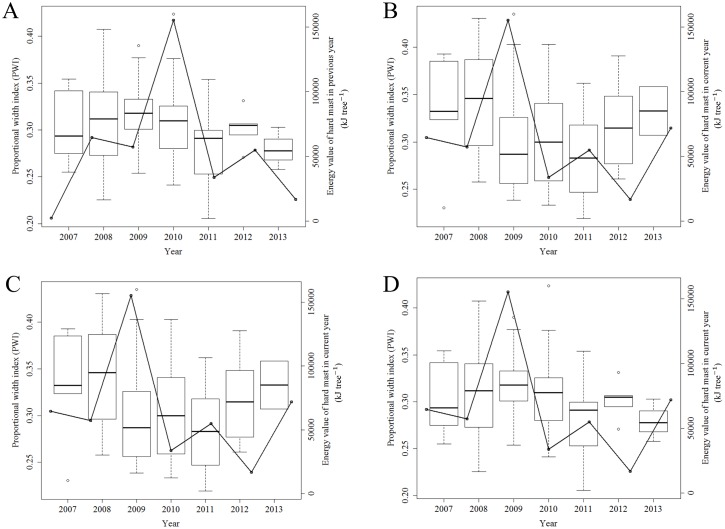
Box plot showing relationships between hard mast production and proportional width index (PWI) of adult or subadult male Asian black bears. (A) Relationship between the PWI in adult period and masting (the averaged energy values (kJ tree^-1^) of hard mast) in previous years. (B) Relationship between the PWI in adult period and masting in current years. (C) Relationship between the PWI in subadult period and masting in previous years. (D) Relationship between the PWI in subadult period and masting in current years.

There was no significant relationship between PWI and the averaged energy values (kJ tree^-1^) from hard mast production each year because the AIC values from both the previous year masting model and the current year masting model were higher than that from the null model for both adult and subadult periods (Tables 3 and 4; Fig 3).

**Table 3:** Linear mixed models showing the effects of hard mast production on proportional width index in male Asian black bears in the adult period.

Fixed effects							
	Variable	Estimate	SE	P-value	95% CI		AIC
Previous masting model	Intercept	0.341	0.058				
	Masting	–0.003	0.005	0.573	–0.014	0.008	–188.4
Current masting model	Intercept	0.385	0.095				
	Masting	–0.007	0.009	0.426	–0.024	0.011	–188.6
Null model	Intercept	0.309	0.009				–190.0
Random effects							
	Group	Variance	SD				
Previous masting model	Individual ID (n = 29)	0.002	0.035				
Current masting model		0.002	0.037				
Null model		0.002	0.043				

**Table 4:** Linear mixed models showing the effects of hard mast production on proportional width index in male Asian black bears in the subadult period.

Fixed effects							
	Variable	Estimate	SE	P-value	95% CI		AIC
Previous masting model	Intercept	0.283	0.025				
	Masting	0.002	0.002	0.367	–0.003	0.007	–425.8
Current masting model	Intercept	0.232	0.060				
	Masting	0.007	0.005	0.223	–0.004	0.017	–426.5
Null model	Intercept	0.305	0.004				–427.0
Random effects							
	Group	Variance	SD				
Previous masting model	Individual ID (n = 51)	0.001	0.023				
Current masting model		0.001	0.023				
Null model		0.001	0.023				

Hard mast production from the previous and current years barely had any impact on cementum annuli widths, as the 95% confidence intervals overlapped zero and the individual differences did not change the effects of hard mast on PWI (Tables 3 and 4).

In our analysis of the false annuli models, the AIC value for the previous year masting model was the lowest of all three models in adult male black bears, but the 95% confidence interval for masting effects included zero (*P* = 0.173); there were therefore no significant effects of hard mast production on the occurrence of false annuli (Table 5).

**Table 5:** Generalized linear mixed models showing the effects of hard mast production on the occurrence of false annuli in male Asian black bears in the adult period.

Fixed effects							
	Variable	Estimate	SE	P-value	95% CI		AIC
Previous masting model	Intercept	–4.953	3.632				
	Masting	0.455	0.033	0.173	–1.414	1.204	75.9
Current masting model	Intercept	5.641	4.413				
	Masting	–0.514	0.402	0.200	–1.361	0.256	76.4
Null model	Intercept	0.000	0.277				76.1
Random effects							
	Group	Variance	SD				
Previous masting model	Individual ID (n = 25)	0.000	0.000				
Current masting model		0.000	0.000				
Null model		0.000	0.000				

In contrast, for subadult period, the AIC value for the previous year masting model was the lowest of all three models; hard mast production from the previous year was negatively associated with false annuli formation, as the 95% confidence interval was below zero (*P* = 0.053) (Table 6).

**Table 6:** Generalized linear mixed models showing the effects of hard mast production on the occurrence of false annuli in male Asian black bears in the subadult period.

Fixed effects							
	Variable	Estimate	SE	P-value	95% CI		AIC
Previous masting model	Intercept	4.082	1.833				
	Masting	–0.329	0.170	0.053	–0.696	–0.014	142.6
Current masting model	Intercept	–2.354	2.071				
	Masting	0.270	0.368	0.464	–0.449	1.021	146.3
Null model	Intercept	0.627	0.242				144.8
Random effects							
	Group	Variance	SD				
Previous masting model	Individual ID (n = 47)	0.223	0.473				
Current masting model		0.306	0.553				
Null model		0.300	0.548				

Larger random effects were seen in the subadult models than in the adult models (Tables 5 and 6); the variability observed in the occurrence of false annuli in the subadult period was therefore affected by individual differences.

## Discussion

Our results showed that there were decreasing width patterns and periods of light- or dark-band formation in the cementum annuli of premolar teeth of Asian black bears, similar to other mammals. In addition, we found that hard mast production had no impact on cementum patterns for adult period, but a small impact on subadult period.

Light bands were formed from May to November whereas dark bands were formed from October to next year of May. These seasons correspond to active and inactive periods for bears throughout the year. It is believed that there are three potential factors causing the formation of annuli in mammals [2]. The first factor is a nutritional change where an increased calcium intake would influence mineralization. The second factor is biomechanical force, meaning that animals chew harder on poorer quality foods during a dry or winter season [2]. The third factor is a change in hormonal cycles that occurs during reproduction. Because bears hibernate during winter [32], their diets change seasonally [33], and females give birth during hibernation [34]. When dark bands are created, the first or third factors are most likely to be true for Asian black bears. In contrast, although there are other factors such as metabolic changes during hibernation [35] and photoperiodicity [2], it is unclear from this study which of these factors are most important.

The annual width (PW) decreases with age, as has been shown in the studies verifying similarly [19, 36, 37]. It is said that fluctuating patterns of annuli widths with age will correspond to growth patterns of body length or the decreasing annuli widths will stop when sexual maturity has been reached [1]. The “break point” of decreasing annuli widths in male bears occurred at about 3.7 years of age. These points do not correspond to the period when body growth stopped (6.1–7.9 years old), but was closer to the period of sexual maturity for male bears (3–4 years old) [38]. Therefore, it is possible that decreasing annuli widths with age can be used as an index for sexual maturity in male Asian black bears as well as in other mammals [1]. In the future, we can establish this index by comparing teeth with the physiological states of individual bears. Notably, the majority of our captured bears were 5 or 6 years old. Young reproductive males may be more likely to be nuisance bears; because of their bold behavior or weaker social status it is likely that they will be found close to human residences.

There was no significant relationship between the calculated cementum annulus index (PWI) and hard mast production for adult male bears. The effects of hard mast production were not reflected in cementum annuli formation because the energy intake from hard mast during the autumn hyperphagia period was used to accumulate fat preferentially rather than to grow bone and muscle [18, 39]. On the basis of the food-switching behavior of bears, we also assumed that because bears are omnivorous feeders, they may feed on hard mast extensively in good mast years, but feed on alternative foods to cover the shortage in poor mast years [40]. Indeed, bears could not gain from alternative foods during poor mast years as much energy as during good mast years [41]. Thus, their nutritional conditions possibly may be lower during poor mast year, but we speculated the masting effect was not so much stronger as reflected in cementum annuli widths. The masting effect had no impacts in either the current year or the previous year; thus, food availability from hard mast production in autumn was not the factor causing cementum annuli widths to become narrower. As a result, we can deduce that reproductive success of female is a strong factor causing a decrease in annuli widths in mature females.

False annuli were observed here in male teeth, as well as in studies of polar bear teeth [11, 25], and the frequency of false annuli formation was significantly different among age classes and only slightly affected by masting. The results of model selection for masting effects on the presence of false annuli in adult males indicated a possibility that false annuli formation was influenced not by food availability, but by other stressful events such as mating [9]. Mating stress can be caused by a combination of attempting to find a mate, fighting with other bears, and not eating during the mating season. In contrast, the results of model selection for the subadult period indicated that masting conditions from previous years have a weak negative effect on false annulus formation, and false annuli form more often in years following a good mast production for subadults. Although the masting effects in current years were not significant, the mean for this parameter showed positive values. Moreover, generally following years of good mast production are years of poor mast production [15]; therefore, for the subadult period, the frequency of false annuli formation may increase in poor mast years. During the early stages of life history, the body is growing rapidly [20, 42], and energy intake, including from hard mast, may be used for the growth of bones and the development of permanent teeth after their eruption; in adult period, this energy is used primarily for fat accumulation. The light bands of cementum generally develop later in younger animals because their body growth periods are longer throughout the year than those growth periods for older bears [1, 43]. Therefore, the probability of false annuli formation is higher in subadults than in adults. Hence, the false annuli of subadults tend to form more frequently, and the formation may be influenced by hard mast production. However, it remains unclear why false annuli form in the cementum of adults. Accordingly, further studies are required to test which factors affect false annuli formation in adult male bears by focusing on stress events such as mating [9].

How and when bears take in food are reflected in the formation of the cementum annuli; therefore, this food intake information is a prerequisite in surveys of the relationship between nutritional status influenced by food availability and cementum annuli formation. In the future, we need to conduct feeding experiments in captive bears to obtain accurate information of this nature. Our results showed that the abundance of food resources in autumn did not influence cementum annuli width in male brown bears; therefore, in females, parturition and lactation are the most likely causes of decreased annuli width.

## Supporting information

S1 File Raw dataset. The dataset of proportional growth layer group width (PW), proportional width index (PWI), presence or absence of false annulus and mast energy of hard mast for each individual and each year.
(ZIP)
